# Ischemia–Reperfusion Injury in Free Flaps: Molecular Mechanisms and Protective Effects of Remote Ischemic Preconditioning

**DOI:** 10.1111/jcmm.70739

**Published:** 2025-08-07

**Authors:** Marius Drysch, Alexander Fiedler, Tabea Kurbacher, Sonja Verena Schmidt, Felix Reinkemeier, Flemming Puscz, Mustafa Becerikli, Maria Fueth, Pia Weskamp, Marcus Lehnhardt, Christoph Wallner, Alexander Sogorski

**Affiliations:** ^1^ Department of Plastic Surgery BG University Hospital Bergmannsheil, Ruhr University Bochum Bochum Germany; ^2^ Department of Gynecology and Obstetrics St. Elisabeth‐Hospital, Ruhr University Bochum Bochum Germany

**Keywords:** apoptosis, breast reconstruction, free flap surgery, ischemia–reperfusion injury, necroptosis, oxidative stress, remote ischemic preconditioning

## Abstract

Ischemia–reperfusion injury remains a major challenge in free flap surgery, contributing to oxidative stress, inflammation, and cell death that impair tissue viability and outcomes. Remote ischemic preconditioning (RIPC) has emerged as a potential protective strategy by modulating cellular stress responses, but its molecular mechanisms in free flaps remain incompletely understood. We prospectively enrolled 36 female patients undergoing autologous breast reconstruction with mainly deep inferior epigastric perforator (DIEP) free flaps, randomised into three groups: No RIPC, Early RIPC (24 h preconditioning), and Late RIPC (1 h preconditioning). Tissue samples were collected pre‐ischemia and post‐reperfusion for immunohistochemical and multiplex protein analyses. RIPC did not reduce oxidative stress markers, as 4‐hydroxynonenal (4‐HNE) levels were comparable across groups, while 3‐nitrotyrosine levels paradoxically increased after RIPC. Early RIPC selectively modulated cell death pathways, with decreased expression of mitochondrial apoptotic marker caspase 9 and reduced necroptotic activation of mixed lineage kinase domain‐like protein (MLKL) after reperfusion. Caspase 8 showed a transient modulation, suggesting effects on apoptosis‐necroptosis crosstalk. Cyclophilin A levels were elevated after reperfusion in RIPC groups, indicating an adaptive stress response. These findings suggest that early RIPC exerts selective protection by modulating apoptosis and necroptosis, rather than broadly reducing oxidative stress. RIPC may represent a targeted strategy to improve free flap survival in reconstructive surgery.

## 1 Introduction

Ischemia–reperfusion (IR) injury remains a major challenge in free flap surgery, contributing to cellular damage, impaired tissue viability, and potential flap failure [1, 2]. The transient ischemia required for flap harvest and transfer leads to a cascade of molecular and physiological disturbances upon reperfusion [3, 4]. While IR injury has been widely recognised in various organ systems, its precise effects on human free flaps remain incompletely characterised [5, 6, 7]. Evidence from other ischemic models suggests that oxidative stress, inflammatory activation, vascular dysfunction, and multiple forms of cell death all contribute to IR‐related tissue damage, but their relative importance in free flaps has yet to be fully elucidated [8, 9, 10, 11, 12, 13]. Understanding these mechanisms is critical for developing targeted protective strategies that can enhance surgical outcomes. Remote ischemic preconditioning (RIPC) has emerged as a promising technique for reducing IR injury by harnessing endogenous protective mechanisms in a remote organ or limb [14, 15]. While RIPC has been extensively studied in cardiac ischemia models, its molecular mechanisms in reconstructive surgery and particularly in free flaps are not well established [16, 17]. Multiple mechanisms have been proposed for RIPC, including humoral signalling, neural pathways, vascular adaptation, and metabolic preconditioning, but their specific roles in flap viability remain unclear [5, 18, 19, 20, 21]. Some preclinical studies suggest that RIPC can enhance microvascular perfusion, reduce inflammatory activation, and modulate cellular stress responses, but its clinical efficacy remains debated, with variable findings in different surgical contexts [5, 16, 19, 21].

At the cellular level, IR injury is characterised by profound metabolic disturbances, oxidative stress, and activation of multiple cell death pathways [6, 9, 12, 22]. While several mechanisms of cell death have been implicated in IR injury, apoptosis, necrosis, and necroptosis are among the most extensively studied and appear to play central roles in determining tissue viability [12, 13, 22, 23]. Apoptosis is a tightly regulated form of programmed cell death that maintains membrane integrity while dismantling cellular components in a controlled manner [24]. It is initiated via either the intrinsic or extrinsic pathways, both culminating in caspase activation [24, 25, 26]. The intrinsic pathway is mitochondria‐dependent and regulated by Bcl‐2 family proteins, where pro‐apoptotic factors such as Bax and Bak facilitate mitochondrial outer membrane permeabilization (MOMP), leading to the release of cytochrome *c*. This triggers apoptosome formation and the activation of caspase 9, which subsequently activates executioner caspases such as caspase 3 [24, 25, 26]. The extrinsic pathway, in contrast, is initiated by death receptors such as tumour necrosis factor receptor (TNF‐R), Fas cell surface death receptor (Fas), or TNF‐related apoptosis‐inducing ligand (TRAIL), leading to caspase 8 activation and downstream effector caspase signalling [27, 28]. Necrosis, in contrast to apoptosis, is an unregulated and catastrophic form of cell death that results in cell lysis, the release of damage‐associated molecular patterns (DAMPs), and extensive inflammation [27, 28, 29]. It occurs when adenosine triphosphate (ATP) depletion, severe oxidative stress, and calcium overload overwhelm cellular homeostasis, leading to loss of plasma membrane integrity. Unlike apoptosis, necrosis is not programmed and lacks molecular checkpoints, making it a major contributor to secondary tissue damage following IR injury. Necroptosis is a regulated form of necrotic cell death that shares features of both apoptosis and necrosis [11, 30]. It is triggered when apoptosis is inhibited, often through caspase 8 inactivation. Necroptosis is mediated by the receptor‐interacting protein kinases receptor‐interacting serine/threonine‐protein kinase 1 (RIPK1) and receptor‐interacting serine/threonine‐protein kinase 3 (RIPK3), which form a necrosome complex that phosphorylates MLKL. Phosphorylated MLKL translocates to the plasma membrane, forming pores that lead to osmotic cell rupture. Necroptotic cell death releases DAMPs such as high‐mobility group box 1 (HMGB1), further exacerbating inflammation. Interestingly, the crosstalk between apoptosis and necroptosis is orchestrated by caspase 8, which, when active, prevents necroptosis by cleaving RIPK1 and RIPK3 [31, 32, 33]. However, when caspase 8 is inhibited, either by viral proteins or ischemic conditions, necrosome formation is promoted, leading to inflammatory cell death [31, 32].

These complex interactions among apoptosis, necrosis, and necroptosis highlight the need for targeted interventions that can mitigate IR injury at multiple levels. By understanding the molecular dynamics of cell death in IR injury, strategies such as RIPC may be optimised to shift the balance toward protective cellular responses and improve free flap viability. This study seeks to refine our understanding of IR injury in human free flaps and explore the translational potential of RIPC as a clinical tool in reconstructive surgery. By characterising the molecular landscape of IR injury, evaluating the potential protective effects of RIPC, and investigating the timing‐dependent impact of preconditioning, we aim to identify key pathways involved in tissue damage and recovery.

To this end, we selected a panel of molecular markers representing the major mechanistic axes implicated in IR injury: apoptosis (caspase 3/8/9, Bcl‐2, BAD), necroptosis (MLKL), oxidative stress (4‐HNE, 3‐nitrotyrosine), and necrosis‐associated damage signalling (HMGB1, cyclophilin A). In addition, we assessed key intracellular signalling molecules involved in the regulation of stress responses and cell fate decisions, specifically p53, JNK and Akt. These pathways are known to integrate IR‐related stimuli and modulate downstream cell death programs. The findings will contribute to a growing body of research aimed at optimising perioperative strategies for improved flap survival and patient outcomes.

## 2 Materials and Methods

### 2.1 Experimental Groups

**FIGURE 1:**
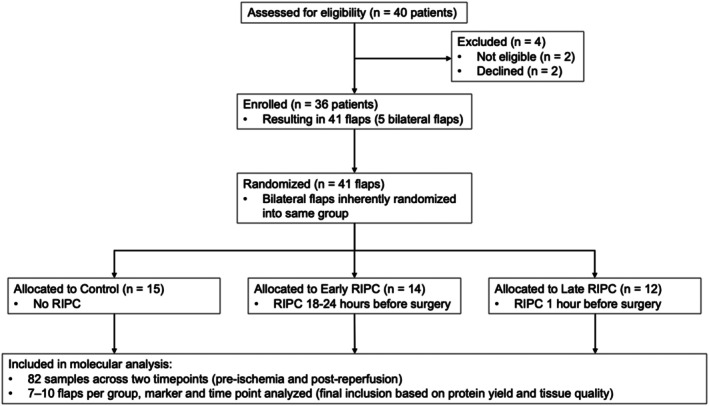
CONSORT flow diagram of patient enrollment, flap randomization and molecular analysis. A total of 40 patients were assessed for eligibility, of whom four were excluded due to ineligibility (*n* = 2) or declining participation (*n* = 2). Thirty‐six patients were ultimately enrolled, including five who underwent bilateral reconstruction, resulting in 41 flaps. Bilateral flaps were inherently assigned to the same intervention group. Flaps were then allocated to one of three groups: Control (No RIPC, *n* = 15), early remote ischemic preconditioning (Early RIPC 18–24 h before surgery, *n* = 14), or Late RIPC (1 h before surgery, *n* = 12). Tissue was collected from each flap at two timepoints: prior to ischemia and after reperfusion. A total of 82 samples were analysed. For each marker and timepoint, 7–10 flaps per group were included in the final analysis, based on tissue quality and protein yield.

Three experimental groups were established to assess the effects of remote ischemic preconditioning (RIPC) on ischemia–reperfusion (IR) injury in free flap surgery (Figure 1). The control group (No RIPC) underwent standard surgical procedures without preconditioning. The RIPC 24 h group (also: Early RIPC) received RIPC 18–24 h before surgery, while the RIPC 1 h group (Late RIPC) underwent RIPC 1 h prior to surgery. Preconditioning was performed using a blood pressure cuff applied to the patient's arm, inflated to 250 mmHg for three cycles of 10‐min occlusion followed by 10‐min reperfusion. Tissue samples were collected before ischemia (pre‐IR, baseline control) and after reperfusion (post‐IR, before final wound closure). The median ischaemia time was 82.0 (60.0–88.0) min, and the median reperfusion time was 70.3 (30.0–90.0) min.

### 2.2 Ethical Approval

This study was conducted in accordance with the Declaration of Helsinki and approved by the Institutional Ethics Committee of the Ruhr University Bochum (Approval Number: 19‐6752, Date: 08.01.2020). All participants provided written informed consent prior to inclusion in the study. All collected samples were anonymized before analysis to ensure compliance with the General Data Protection Regulation (GDPR) and institutional guidelines on patient data protection. Patients were randomly assigned to one of three study groups (No RIPC, Early RIPC and Late RIPC). The study protocol was reviewed and approved by the ethics committee to ensure adherence to guidelines for clinical research involving human participants.

### 2.3 Immunofluorescence

Snap‐frozen tissue samples were cryosectioned (7 μm thick) and fixed in acetone (10 min at −20°C) for membrane permeabilization. Sections were circled with a hydrophobic barrier pen and subjected to blocking (2 h, phosphate‐buffered saline [PBS] with host species serum) before overnight incubation (4°C) with the following primary antibodies: MLKL (rabbit monoclonal, 1:100; Abcam, ab184718), HMGB1 (rabbit polyclonal, 1:50; Abcam, ab18256), Caspase 3 (rabbit polyclonal, 1:50; Santa Cruz, sc‐7148), 3‐Nitrotyrosine (mouse monoclonal, 1:300; Abcam, ab61392), 4‐Hydroxynonenal (mouse monoclonal, 1:100; R&D Systems, MAB3249), Cyclophilin A (rabbit polyclonal, 1:200; Abcam, ab3563), and Myeloperoxidase (goat polyclonal, 1:100; R&D Systems, AF3667). Following primary incubation, sections were washed in PBS, incubated with fluorophore‐conjugated secondary antibodies (2 h, room temperature), and counterstained with DAPI mounting medium. Images were acquired on a Zeiss Axio Vert A microscope using consistent exposure settings for each marker. For quantification, at least three random, non‐overlapping high‐power fields were analysed per sample. Analysis was performed in Adobe Photoshop using the Magic Wand tool (tolerance 20%), a setting empirically determined to best separate signal from background. To eliminate inter‐observer variability, all quantifications were performed by a single investigator blinded to the experimental groups. Fluorescence‐positive pixel counts were normalised to the number of DAPI‐stained nuclei per field. Figure images are representative of the mean for each group.

### 2.4 Multiplex Protein Analysis (Luminex Assay)

Tissue lysates were prepared in RIPA buffer. Samples were retrieved from −80°C storage, placed on ice, and homogenised using a rotor‐stator homogeniser. Additional RIPA buffer (300 μL) was added, followed by further homogenization. Lysates were incubated on an orbital shaker (4°C, 2 h) and centrifuged (15,000 *g*, 15 min, 4°C). Supernatants were collected, and total protein concentrations were quantified using a bicinchoninic acid (BCA) assay (Thermo Fisher; Cat# 23225). Samples were stored at −80°C until further analysis.

A MILLIPLEX Early Apoptosis Magnetic Bead 7‐Plex Kit (Merck) was used to quantify apoptotic protein levels. Protein samples were normalised to a uniform concentration and diluted 1:5 in assay buffer. The assay was conducted according to manufacturer instructions, and fluorescence signals were detected using a Luminex xMAP system. Data were analysed using GraphPad Prism.

### 2.5 Statistical Analysis

All statistical analyses were performed using Python (Version 3.10) with SciPy, StatsModels, and Matplotlib libraries and GraphPad Prism (Version 10.3.0; GraphPad Software, San Diego, CA, USA). Data were assessed for normality using the Shapiro–Wilk test. Depending on data distribution, comparisons between two groups were conducted using an unpaired two‐tailed Student's *t*‐test (for normally distributed data) or the Mann–Whitney *U* test (for non‐normally distributed data). For multiple group comparisons, a two‐way ANOVA with Tukey's post hoc test was used for parametric data, while the Kruskal–Wallis test with Dunn's post hoc correction was applied for non‐parametric data. Data are presented as mean ± standard error of the mean (SEM) for immunofluorescence quantifications, as mean ± standard deviation (SD) for normally distributed demographic data as BMI and age, and as median with interquartile range (IQR) for non‐normally distributed variables such as ischemia, reperfusion, and total surgery time. Where applicable, box‐and‐whisker plots display the median, IQR, and minimum‐to‐maximum values. A significance threshold of *p* < 0.05 was considered statistically significant, with additional thresholds indicated as *p* < 0.01 and *p* < 0.001 where applicable.

### 2.6 Correlation and Regression Analyses

To evaluate the relationship between molecular markers (e.g., c‐Jun N‐terminal kinase [JNK], Bcl‐2‐associated agonist of cell death [BAD], B‐cell lymphoma 2 [BCL‐2], protein kinase B [AKT], caspase 9, p53, caspase 8) and clinical parameters (ischemia time, reperfusion time, and total surgery time), Pearson's correlation coefficient was used for parametric data and Spearman's rank correlation coefficient for non‐parametric data. Correlations were assessed separately for each experimental group (No RIPC, Early RIPC and Late RIPC). To determine whether group differences influenced molecular marker expression, an analysis of covariance (ANCOVA) was performed, including ischemia time, reperfusion time, and total surgery time as covariates, with experimental group as the independent variable. For graphical representation, scatter plots with fitted trend lines were generated for each molecular marker against ischemia time, reperfusion time and total surgery time. Data points were color‐coded for each experimental group (No RIPC: blue, Early RIPC: orange, Late RIPC: green), and linear regression lines were fitted to illustrate potential trends.

## 3 Results

### 3.1 Overview of Patient Demographics

A total of 41 flaps (Table 1) were included in the study, all of whom were from female patients (100%). The mean age of the cohort was 48.2 ± 8.6 years, and the mean body mass index (BMI) was 27.41 ± 4.6 kg/m^2^. All patients underwent autologous breast reconstruction with a free flap (100%). The most frequently used flap was the unilateral deep inferior epigastric artery perforator (DIEP) flap, performed in 26 patients (63.4%), followed by bilateral DIEP in 10 patients (24.4%). Other flap types included the muscle‐sparing transverse rectus abdominis muscle (ms‐TRAM) flap (7.3%), profunda artery perforator (PAP) flap (2.4%), and transverse myocutaneous gracilis (TMG) flap (2.4%). The median ischemia time was 82.0 (60.0–88.0) min, while the median reperfusion time was 70.3 (30.0–90.0) min. The median total surgery duration was 412.1 (352.5–500) min.

**TABLE 1:** Overview of patient and flap characteristics.

Characteristic	
Female (%)	41 (100%)
BMI (±SD)	27.41 (±4.6)
Age (±SD)	48.2 (±8.6)
Flap (%)	41 (100%)
Unilateral DIEP (%)	26 (63.4%)
Bilateral DIEP (%)	10 (24.4%)
ms‐TRAM (%)	3 (7.3%)
PAP (%)	1 (2.4%)
TMG (%)	1 (2.4%)
Ischemia time (IQR)	82.0 (60.0–88.0)
Reperfusion time (IQR)	70.3 (30.0–90.0)
Total surgery time (IQR)	412.1 (352.5–500)

### 3.2 RIPC Enhances Nitrosative Stress Without Reducing Oxidative Damage

**FIGURE 2:**
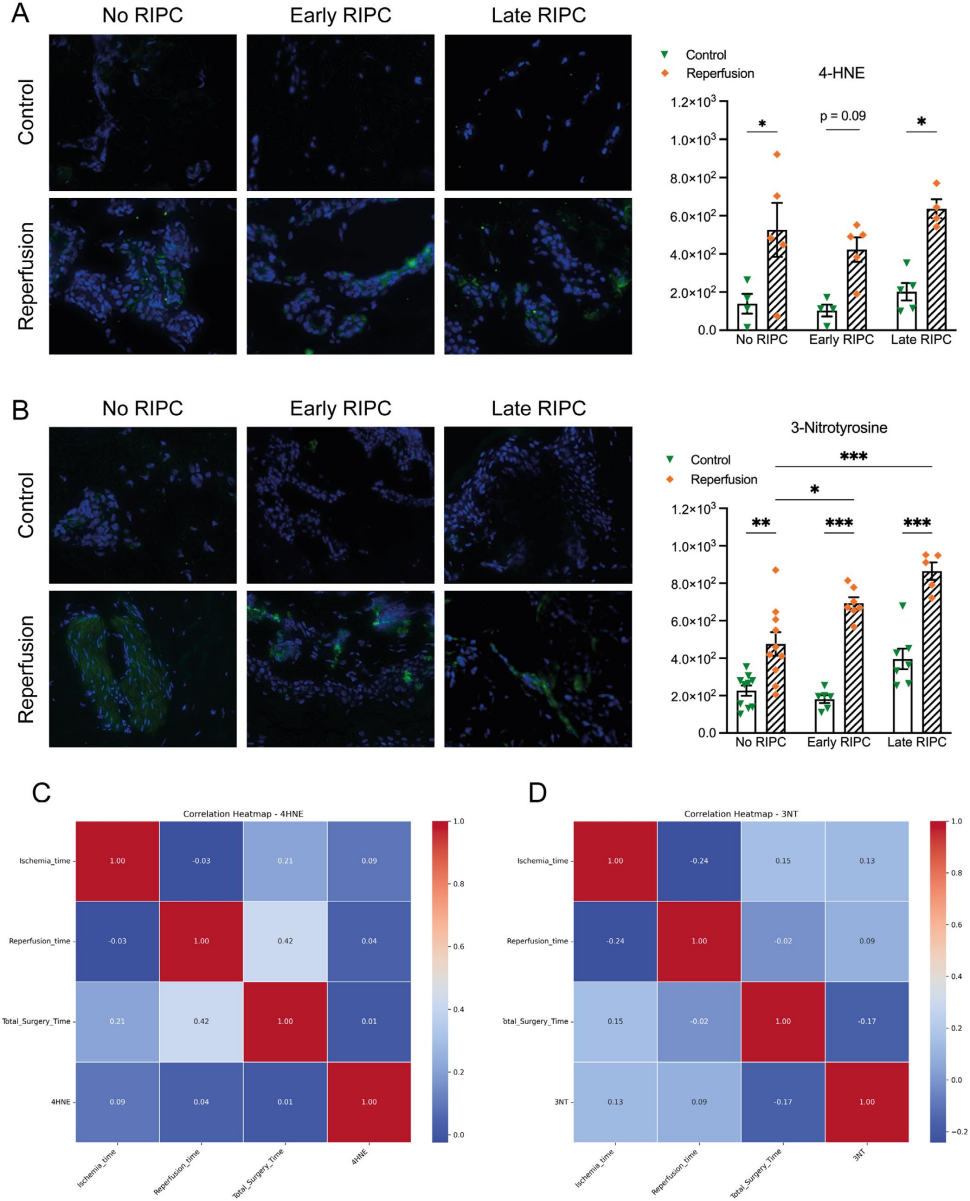
Immunofluorescence analysis of lipid peroxidation and protein nitration. (A) Representative images of 4‐HNE staining (green), a marker of lipid peroxidation, in control and reperfusion conditions across No RIPC, Early RIPC and Late RIPC groups. Quantification shows increased 4‐HNE accumulation post‐reperfusion in all groups, with no significant differences between RIPC‐treated and untreated conditions. (B) Representative images of 3‐NT staining (green), a marker of protein nitration. Post‐reperfusion quantification indicates significantly higher 3‐NT levels in both RIPC groups compared to No RIPC, suggesting RIPC enhances nitrosative stress. (C, D) Correlation matrices illustrating the relationships between ischemia time, reperfusion time, total surgery time and post‐reperfusion levels of 4‐HNE (C) and 3‐NT (D). No significant correlations were observed. Data are presented as mean ± SEM. *p < 0.05, **p < 0.01, ***p < 0.001.

IR injury is known to induce oxidative stress, as reflected in the accumulation of lipid peroxidation and protein nitration markers. To evaluate whether RIPC modulates oxidative stress levels, we assessed 4‐hydroxynonenal (4‐HNE) and 3‐nitrotyrosine (3‐NT) expression across different conditions. 4‐HNE as a marker of lipid peroxidation exhibited a general increase following IR injury across all groups, but the effect of RIPC was limited. In the No RIPC group, 4‐HNE levels increased 2.5‐fold (*p* = 0.03) post‐reperfusion compared to baseline, reflecting expected oxidative stress following IR injury. The Late RIPC group showed a similar trend, with a 3.1‐fold increase after reperfusion (*p* = 0.01), while the Early RIPC group exhibited a 4.2‐fold rise (*p* = 0.09). However, despite these increases, there were no significant differences between the RIPC groups and No RIPC in terms of 4‐HNE accumulation (*p* > 0.05 in all comparisons). This suggests that RIPC, regardless of timing, did not substantially alter lipid peroxidation dynamics in response to IR injury. Similar to 4‐HNE, 3‐NT levels, as a marker of protein nitration, increased significantly post‐IR in all groups, confirming the expected oxidative stress response to ischemia–reperfusion. However, while 4‐HNE accumulation did not differ significantly between groups, 3‐NT levels were significantly higher in both RIPC groups post‐IR when compared to the No RIPC group (1.45‐fold, *p* < 0.05, and 1.86‐fold, *p* < 0.001), suggesting a potential RIPC‐induced modulation of nitric oxide‐related pathways. Given the known susceptibility of oxidative stress markers to time‐dependent metabolic changes, we conducted correlation analyses between ischemia time, reperfusion time and total surgery time with post‐IR 4‐HNE and 3‐NT levels. However, no significant correlations were observed (Figure 2).

### 3.3 RIPC Attenuates IR‐Induced Apoptosis in a Time‐Dependent Manner

**FIGURE 3:**
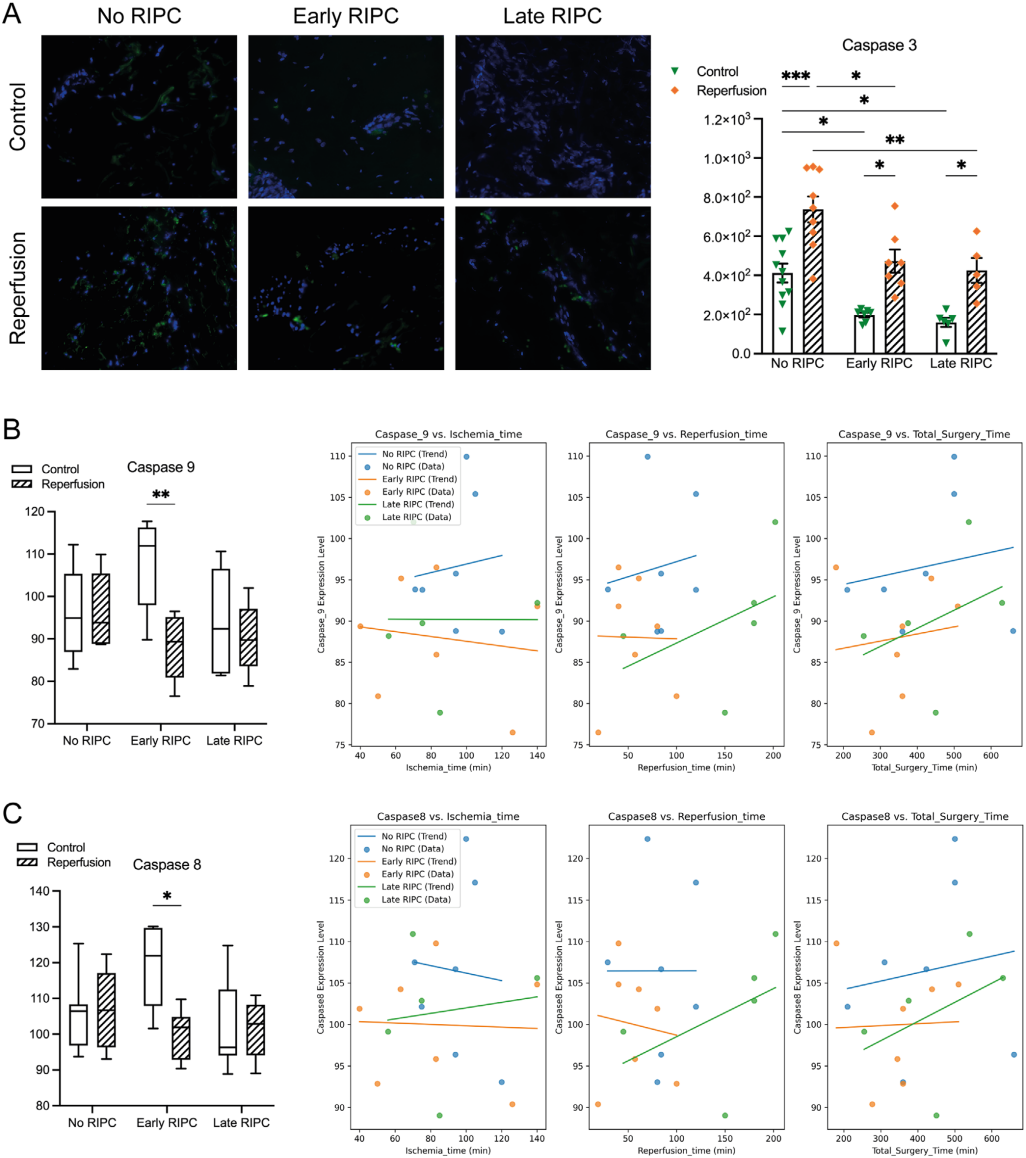
Analysis of apoptotic markers. (A) Representative immunofluorescence images of caspase 3 staining (green) in control and reperfusion conditions across No RIPC, Early RIPC, and Late RIPC groups. Quantification demonstrates a significant post‐reperfusion increase in caspase 3 levels in the No RIPC group, while both Early and Late RIPC groups exhibit significantly lower caspase 3 expression, suggesting a protective effect of RIPC against apoptosis. (B) Caspase 9 expression pre‐ and post‐reperfusion across all groups. Caspase 9 levels remained unchanged in the No RIPC and Late RIPC groups but were significantly lower post‐reperfusion in the Early RIPC group, indicating suppression of intrinsic (mitochondrial) apoptotic signalling. Scatter plots depict the correlation of caspase 9 levels with ischemia time, reperfusion time, and total surgery time. (C) Caspase 8 expression pre‐ and post‐reperfusion across all groups, demonstrating a moderate pre‐IR increase in the Early RIPC group, followed by a significant post‐reperfusion decline. Scatter plots illustrate the correlation of caspase 8 levels with ischemia time, reperfusion time and total surgery time. Data are presented as mean ± SEM for immunofluorescence quantifications. Box plots represent the median, IQR, and minimum‐to‐maximum values. *p < 0.05, **p < 0.01, ***p < 0.001.

Ischemia–reperfusion (IR) injury is a well‐established trigger of apoptosis, and RIPC has been proposed as a modulator of apoptotic pathways. To assess its impact, we analysed key markers of extrinsic and intrinsic apoptosis (Figure 3). Caspase 3, the main executioner caspase in apoptosis, exhibited a significant increase post‐reperfusion in the No RIPC group, underscoring the robust apoptotic response to IR injury. Both Early and Late RIPC groups displayed lower baseline caspase 3 levels (0.48‐fold, *p* = 0.04 and 0.38‐fold, *p* = 0.02, respectively), suggesting an initial downregulation of apoptotic activity prior to IR. Following reperfusion, caspase 3 levels remained significantly reduced in both RIPC groups (0.54‐fold, *p* = 0.01 and 0.63‐fold, *p* = 0.005, respectively), supporting a protective trend associated with preconditioning. Caspase 8, a key initiator of extrinsic apoptosis, demonstrated a moderate increase in the Early RIPC group pre‐IR (1.13‐fold vs. No RIPC, *p* = 0.05), suggesting a potential priming effect of preconditioning. However, following reperfusion, caspase 8 levels significantly declined in the Early RIPC group (0.84‐fold vs. pre‐IR, *p* = 0.02), indicating a suppression of extrinsic apoptotic signalling. No substantial changes were observed in the No RIPC or Late RIPC groups. Caspase 9, the initiator caspase of the intrinsic (mitochondrial) apoptotic pathway, remained unchanged post‐reperfusion in the No RIPC and Late RIPC groups, but was significantly lower in the Early RIPC group compared to its pre‐IR levels (0.87‐fold, *p* = 0.009). This finding further supports the hypothesis that early RIPC exerts protective effects on mitochondrial apoptosis following IR injury. Regarding the interplay with clinical parameters, caspase 8 and caspase 9 levels exhibited a marginal decline with increasing ischemia and reperfusion times in the Early RIPC group. While not statistically significant, this trend may indicate that longer ischemic stress in the presence of preconditioning could influence apoptotic regulation over time. The biological relevance of this observation remains to be fully elucidated, but it highlights the potential for subtle temporal interactions between preconditioning and IR‐related apoptosis.

### 3.4 Anti‐Apoptotic Effects of RIPC Are Conveyed Independently of Sustained Bcl‐2 Activation

**FIGURE 4:**
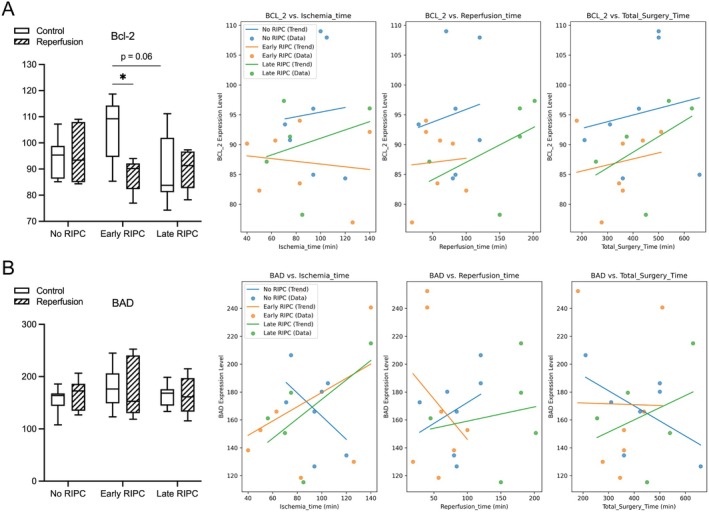
Analysis of Bcl‐2 family proteins. (A) Bcl‐2 expression pre‐ and post‐reperfusion across No RIPC, Early RIPC, and Late RIPC groups. A trend toward higher Bcl‐2 expression was observed in the Early RIPC group pre‐IR compared to Late RIPC (*p* = 0.06), suggesting a potential priming effect. Following reperfusion, Bcl‐2 levels in the Early RIPC group significantly decreased compared to pre‐IR values (*p* = 0.02), while remaining comparable to other groups post‐IR. The No RIPC and Late RIPC groups exhibited stable Bcl‐2 levels with no significant changes. Scatter plots depict the correlation of Bcl‐2 expression with ischemia time, reperfusion time and total surgery time. (B) BAD expression pre‐ and post‐reperfusion across No RIPC, Early RIPC, and Late RIPC groups. No significant differences were observed between control and post‐IR conditions, indicating that BAD does not play a major role in apoptosis regulation in this setting. Scatter plots show no clear correlations between BAD levels and ischemia, reperfusion, or total surgery time. Data are presented as mean ± SEM for immunofluorescence quantifications. Box plots represent the median, interquartile range (IQR), and minimum‐to‐maximum values. *p < 0.05.

Next, we wanted to further elucidate the role of Bcl‐2 family proteins in apoptotic regulation following IR injury. Given their critical function in mitochondrial integrity and apoptosis control, we analysed whether a potential RIPC‐mediated protection could be associated with shifts in the balance between pro‐apoptotic and anti‐apoptotic Bcl‐2 family members (Figure 4). Bcl‐2 showed a trend toward higher expression in the Early RIPC versus Late RIPC before IR (*p* = 0.06), suggesting a potential priming effect of prolonged preconditioning. Following reperfusion, Bcl‐2 levels in this group decreased compared to pre‐IR levels (*p* = 0.02), while remaining within a comparable range to the other groups. In contrast, the No RIPC and Late RIPC groups exhibited more stable Bcl‐2 levels, with no significant changes between pre‐ and post‐IR measurements. This pattern suggests that Early RIPC may transiently enhance anti‐apoptotic signalling, with potential dynamic regulation in response to IR. BAD, a pro‐apoptotic Bcl‐2 family protein, remained unchanged across all groups, with no significant differences observed between control and post‐IR samples. This suggests that BAD does not play a major role in apoptosis regulation in this setting. Exploring procedural influences, Bcl‐2 levels exhibited a slight upward trend with longer ischemia and reperfusion times, suggesting a possible time‐dependent adaptation in anti‐apoptotic signalling. However, these correlations remained modest and did not reach statistical significance. Taken together, these findings suggest that Early RIPC provides the most pronounced anti‐apoptotic effect, particularly through suppression of mitochondrial apoptosis, as evidenced by the significant reduction in caspase 9 levels post‐reperfusion (see Figure 2). However, this protective effect does not appear to be mediated by persistent Bcl‐2 upregulation. Instead, the transient activation and subsequent suppression of caspase 8 in the Early RIPC group (see Figure 3) suggest a more complex regulatory mechanism, potentially involving early priming of apoptotic pathways that ultimately leads to protection against sustained apoptosis post‐reperfusion.

### 3.5 Early RIPC Modulates Necroptosis via MLKL

**FIGURE 5:**
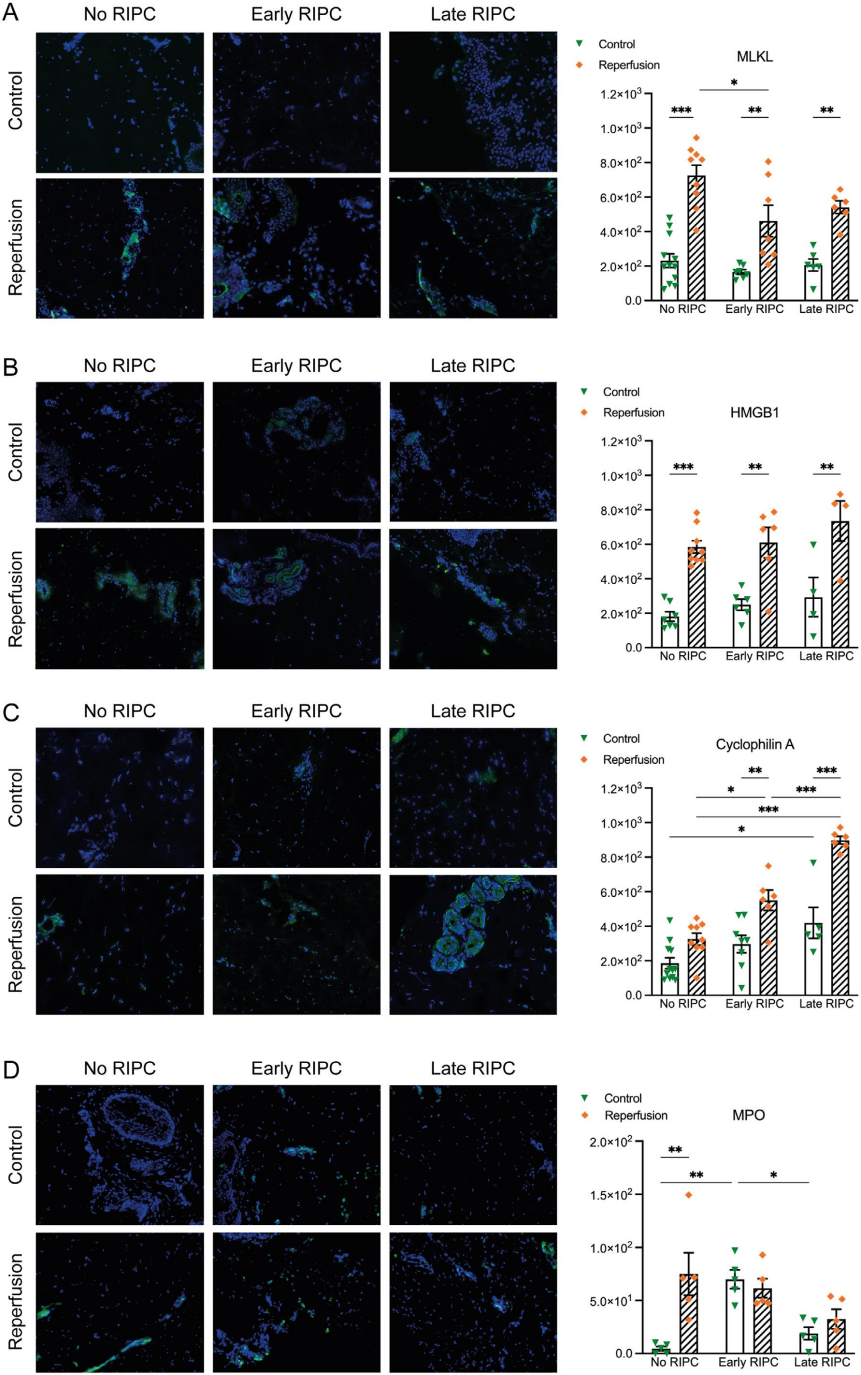
Influence of RIPC on necrosis and necroptosis. (A) Representative immunofluorescence images of MLKL staining (green) in control and reperfusion conditions across No RIPC, Early RIPC, and Late RIPC groups. MLKL expression was significantly increased post‐reperfusion in all groups, confirming necroptotic activation following IR injury. However, MLKL levels were significantly lower in the Early RIPC group compared to No RIPC (*p* < 0.05), while Late RIPC showed a similar but non‐significant trend. (B) Representative immunofluorescence images of HMGB1 staining (green) across all groups. Post‐reperfusion, HMGB1 levels were significantly elevated in all groups, indicating necrotic cell damage and DAMP release. No significant differences were observed between RIPC groups and No RIPC, suggesting that RIPC does not substantially alter HMGB1‐mediated necrotic signalling. (C) Representative immunofluorescence images of cyclophilin A (CyPA) staining (green). CyPA levels significantly increased post‐reperfusion in both Early (*p* < 0.01) and Late RIPC (*p* < 0.001) groups but not in the No RIPC group. Additionally, post‐IR CyPA levels were significantly higher in both RIPC groups compared to No RIPC (*p* < 0.05 and *p* < 0.001, respectively), suggesting a distinct regulatory pattern, potentially reflecting mitochondrial stress adaptation rather than direct necrotic injury. (D) Representative immunofluorescence images of myeloperoxidase (MPO) staining (green) as a surrogate for neutrophil infiltration. MPO levels increased significantly post‐reperfusion in the No RIPC group (*p* < 0.01), while both Early and Late RIPC groups showed no significant post‐reperfusion increase. Interestingly, baseline MPO levels were markedly higher in the Early RIPC group compared to No RIPC (*p* < 0.01), but no further increase occurred post‐reperfusion, suggesting a preconditioned attenuation of neutrophil response. Data are presented as mean ± SEM for immunofluorescence quantifications. *p < 0.05, **p < 0.01, ***p < 0.001.

Beyond apoptosis, necroptosis represents another regulated form of cell death that contributes to IR injury. To evaluate the extent of necrosis and necroptosis following IR injury, we analysed the expression (Figure 5) of necrosis‐associated mediators mixed lineage kinase domain like pseudokinase (MLKL), high‐mobility group box 1 protein (HMGB1), and cyclophilin A (CyPA), which serve as key mediators of regulated necrosis and cellular damage‐associated molecular patterns (DAMPs). MLKL, a key mediator of necroptosis, was significantly upregulated after reperfusion in all groups (*p* < 0.001, No RIPC; *p* < 0.01, RIPC 1 h; *p* < 0.01, RIPC 24 h). Pre‐IR, MLKL levels were comparable across groups, indicating no significant baseline differences in necroptotic signalling. Following reperfusion, MLKL expression was lower in both RIPC groups compared to the No RIPC group, with a significant reduction in the Early RIPC group (*p* < 0.05), while Late RIPC showed a similar but non‐significant trend. This pattern indicates a potential time‐dependent modulation of necroptosis by RIPC, with the Early RIPC protocol exerting a more pronounced inhibitory effect on necroptotic activation following ischemia–reperfusion injury. HMGB1, a nuclear protein passively released from necrotic cells and a potent pro‐inflammatory mediator, showed a significant increase after reperfusion in all groups (*p* < 0.001, No RIPC; *p* < 0.01, RIPC 1 h; *p* < 0.01, RIPC 24 h). No significant differences were observed between the RIPC groups and No RIPC, indicating that RIPC did not substantially alter HMGB1 release. Cyclophilin A followed a distinct pattern. While post‐reperfusion CyPA levels increased significantly in the Early (*p* < 0.01) and Late RIPC (*p* < 0.001) groups, no significant increase was observed in the No RIPC group. Additionally, CyPA levels in both groups were significantly increased post‐IR when compared to the No RIPC group (*p* < 0.05 and *p* < 0.001). This selective increase suggests a potentially different mechanism underlying CyPA dynamics. Given its established role in mitochondrial stress responses, the higher CyPA levels in Early and Late RIPC groups may reflect an adaptive response rather than direct necrotic injury. Notably, the Early RIPC group exhibited the highest absolute levels, possibly indicating a transient stress adaptation associated with short‐term preconditioning rather than overt necrotic damage. In addition to molecular markers of necroptosis and DAMP release, we evaluated myeloperoxidase (MPO) as a surrogate for neutrophil infiltration and acute inflammation (Figure 5). MPO levels rose significantly after reperfusion in the No RIPC group (*p* < 0.01), reflecting a robust neutrophilic response to IR injury. In contrast, both Early and Late RIPC groups showed no significant increase in MPO following reperfusion, suggesting that RIPC attenuates neutrophil recruitment. Notably, baseline MPO levels were markedly higher in the Early RIPC group compared to No RIPC (*p* < 0.01 and *p* < 0.05), possibly reflecting a primed inflammatory state induced by preconditioning. However, despite this elevated baseline, no further MPO elevation occurred post‐reperfusion in the Early RIPC group, supporting the interpretation of an attenuated inflammatory response. These findings suggest that RIPC not only modulates necroptotic signalling but also dampens downstream leukocyte activation.

### 3.6 RIPC Modulates JNK and p53 but Does Not Alter Akt Survival Signalling

**FIGURE 6:**
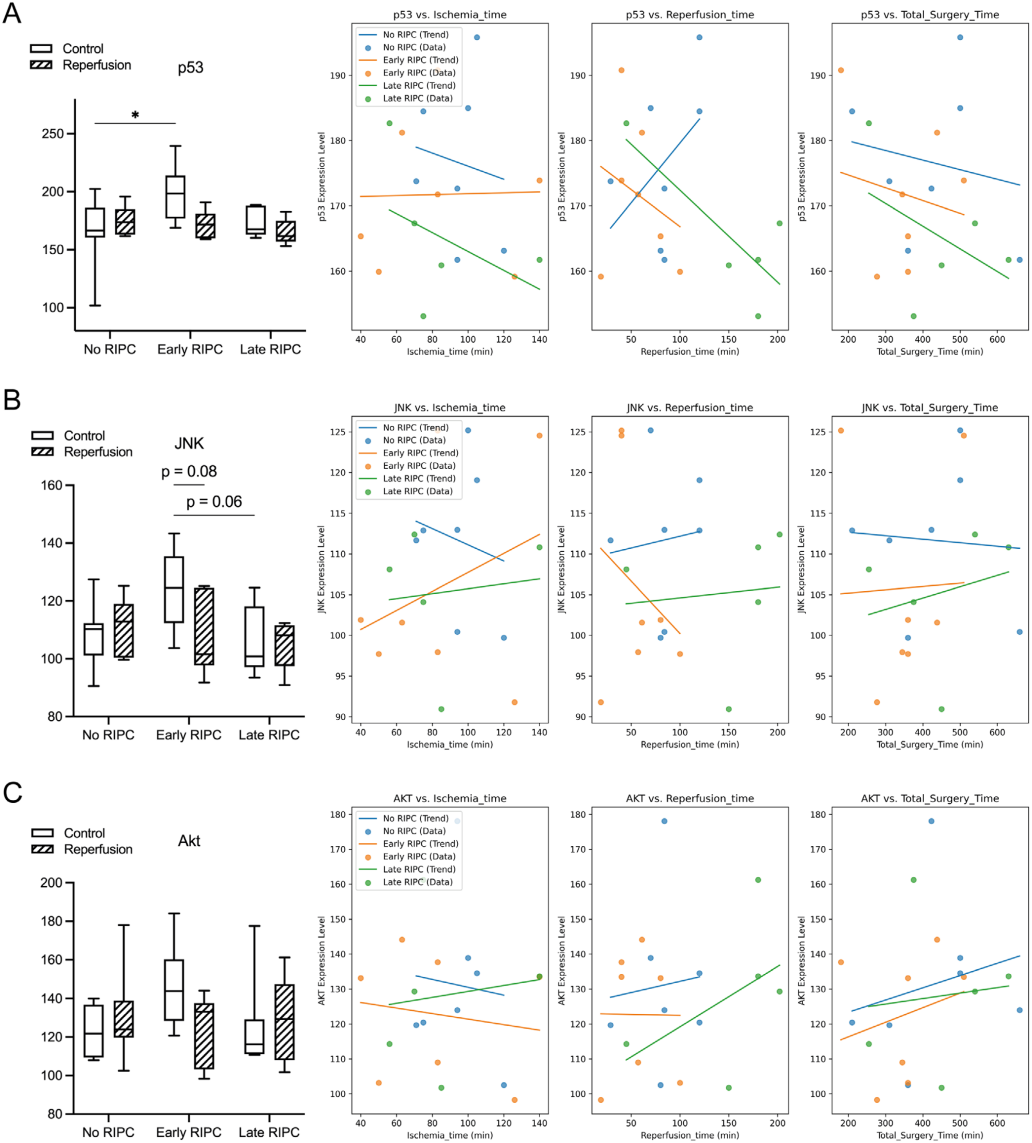
Effects of RIPC on intracellular signalling. (A) p53 expression pre‐ and post‐reperfusion across No RIPC, Early RIPC, and Late RIPC groups. A significant pre‐IR increase in p53 levels was observed in the Early RIPC group compared to No RIPC (*p* = 0.04), suggesting a potential shift toward cell cycle arrest or apoptosis regulation following prolonged preconditioning. However, within‐group differences between control and reperfusion conditions remained non‐significant. (B) JNK expression pre‐ and post‐reperfusion. A trend toward increased JNK levels was observed pre‐IR in the Early RIPC group compared to No RIPC (*p* = 0.06), with a near‐significant decrease post‐IR (*p* = 0.08), suggesting a possible transient stress response modulated by RIPC. (C) Akt expression pre‐ and post‐reperfusion. Akt levels exhibited considerable variability across groups, with no significant differences observed between conditions. This indicates that neither RIPC nor IR had a dominant regulatory effect on Akt‐mediated pro‐survival signalling. Data are presented as box‐and‐whisker plots showing the median, IQR and minimum‐to‐maximum values. *p < 0.05.

To further elucidate the molecular mechanisms underlying RIPC's effects on IR injury, we examined key intracellular signalling pathways involved in stress response and cell survival (Figure 6). JNK, a central kinase in stress‐induced apoptosis and inflammation, exhibited a trend toward increased levels pre‐IR in the Early RIPC group compared to the No RIPC group (*p* = 0.06), with a near‐significant decrease post‐IR (*p* = 0.08). Levels of p53 as master regulator of cell fate demonstrated a significant increase pre‐IR in the Early RIPC group compared to No RIPC (*p* = 0.04), indicating a potential shift toward cell cycle arrest or apoptosis regulation following extended preconditioning. However, within‐group differences between control and reperfusion samples remained non‐significant. Akt, a crucial pro‐survival kinase, displayed considerable variability across conditions, with no significant differences observed between groups or within control‐reperfusion comparisons. This suggests that neither RIPC nor IR exerted a dominant regulatory effect on Akt‐mediated pro‐survival signalling. Taken together, these findings indicate that JNK and p53 signalling may be moderately influenced by RIPC, while Akt levels remained largely unaffected, suggesting that survival signalling was not substantially altered under these conditions.

## 4 Discussion

IR injury is a critical challenge in free flap surgery, with oxidative stress, apoptosis, and necroptosis contributing to cellular damage and impaired tissue viability [1, 2, 14, 21]. While RIPC has been extensively studied in animal models, its mechanistic effects in human tissue remain underexplored [34, 35, 36, 37]. This study provides novel insights into the molecular events underlying IR injury and the impact of RIPC, emphasising selective modulation of apoptosis and necroptosis rather than broad cytoprotection. These findings refine our understanding of RIPC's therapeutic potential and challenge conventional assumptions regarding its mechanisms of action.

Oxidative stress is a well‐established mediator of IR injury, with ischemic conditioning strategies presumed to mitigate ROS‐induced damage [2, 8, 23, 34]. However, our results indicate that RIPC did not significantly alter lipid peroxidation (4‐HNE) levels, suggesting that oxidative stress in human free flaps may be less amenable to RIPC modulation than previously assumed. In contrast, protein nitration, as measured by 3‐NT levels, increased in both RIPC groups, revealing a counterintuitive effect. Rather than broadly reducing oxidative stress, RIPC may instead influence nitric oxide‐related pathways, potentially through enhanced peroxynitrite formation due to transient mitochondrial stress or an adaptive shift in redox signalling, for example, via upregulation of endothelial nitric oxide synthase (eNOS) during ischemia [4]. This suggests that RIPC induces a selective redox response rather than a uniform suppression of oxidative damage. Further studies should determine whether the 3‐NT increase enhances cellular resilience or represents an unintended consequence of RIPC. The lack of significant changes in oxidative stress markers contrasts with preclinical studies in cardiac and cerebral IR models, where RIPC has been shown to upregulate antioxidant defence mechanisms [38, 39]. Several factors may explain this discrepancy: species‐specific differences in redox homeostasis, variability in tissue metabolism and ischemic tolerance, and the potential for delayed antioxidant responses that were not captured within the study's timeframe. Our findings emphasise the need for a more nuanced understanding of oxidative stress regulation in surgical settings and suggest that apoptosis and necroptosis may be more relevant therapeutic targets for RIPC in flap surgery. Notably, these results align with recent clinical data suggesting that while oxidative stress remains a hallmark of IR injury, positive effects of oxidative stress modulation in humans may be inherently limited [40, 41].

Apoptosis and necroptosis are key determinants of flap survival following IR injury. Our findings reveal a timing‐dependent effect of RIPC, with Early RIPC exerting the most pronounced influence by regulating apoptotic and necroptotic pathways. Specifically, Early RIPC significantly reduced caspase 9 activation post‐reperfusion, suggesting enhanced mitochondrial integrity and protection against intrinsic apoptosis. Of note, Lv et al. [39] demonstrated the same mechanistic effect of RIPC in a murine stroke model. Simultaneously, MLKL suppression in Early RIPC indicates an inhibitory effect on necroptotic signalling, suggesting that RIPC actively mitigates both regulated cell death pathways. Complementing these findings, our analysis of neutrophil infiltration via MPO staining revealed a similarly complex, time‐dependent effect. A particularly intriguing finding was the significantly elevated baseline MPO level in the Early RIPC group, observed 18–24 h after the conditioning stimulus. This suggests a systemic ‘priming’ response, where the initial remote event may trigger a slow mobilisation of neutrophils, leading to a higher number of leukocytes in the peripheral tissue before the main surgery. Despite this higher starting point, the inflammatory response to the IR insult was effectively blunted in this group, as evidenced by the lack of a further significant increase in MPO. This supports a dual role for Early RIPC: it modulates the baseline immune state and confers protection against acute inflammatory triggers.

Caspase 8, a key switch between apoptosis and necroptosis, exhibited a transient activation pre‐IR in Early RIPC, followed by a significant decline post‐reperfusion. This pattern suggests that preconditioning may modulate apoptotic susceptibility while also preventing necroptotic initiation. Given that caspase 8 actively inhibits necroptosis by cleaving RIPK1 and RIPK3, its early activation could serve as a regulatory mechanism, reducing the likelihood of a shift toward necroptosis while maintaining apoptotic pathway regulation [11]. The absence of this effect in Late RIPC highlights the importance of timing in preconditioning, suggesting that a sufficient interval prior to ischemia may be required to establish this regulatory mechanism. However, the exact functional implications of this transient caspase 8 activation remain to be fully elucidated, and further studies are needed to determine whether this priming effect translates into a significant protective advantage. Interestingly, Bcl‐2 levels were not persistently elevated, indicating that RIPC‐mediated apoptosis suppression occurs independently of traditional anti‐apoptotic signalling. This diverges from ischemic conditioning models in myocardial IR injury, where sustained Bcl‐2 upregulation is a hallmark of protection [42, 43]. The interplay between caspase 8 priming, caspase 9 suppression, and MLKL inhibition suggests that RIPC dynamically regulates apoptotic and necroptotic thresholds rather than merely inhibiting cell death, a novel concept that may have clinical implications for optimising flap viability.

The role of intracellular signalling in IR injury and RIPC remains under investigation. Our findings challenge the assumption that ischemic conditioning broadly enhances pro‐survival pathways, as RIPC did not significantly alter Akt signalling [44, 45]. Instead, JNK and p53 modulation suggest an alternative regulatory mechanism. Pre‐IR JNK elevation in Early RIPC indicates a potential adaptive stress response, while transient p53 upregulation may reflect cytostatic priming rather than direct apoptotic inhibition [46, 47]. This aligns with cardiology models linking stress kinase activation to ischemic tolerance [48]. The lack of sustained Akt activation reinforces that RIPC fine‐tunes stress‐responsive pathways rather than broadly promoting cell survival, underscoring its selective cytoprotective effects.

Despite novel insights, this study has several limitations that should be considered. Firstly, our findings may have limited generalizability as the study population consisted exclusively of female patients within a defined age and BMI range. Given that factors like sex hormones and metabolic state can influence IRI and conditioning responses, further research is required to validate these mechanisms in male patients and broader patient demographics. Secondly, our analysis was restricted to a single post‐reperfusion time point taken before final wound closure. This provides a valuable snapshot of early molecular events but does not capture the full temporal dynamics of cell death and repair, which can evolve over hours or days. Lastly, our cohort was composed predominantly of adipocutaneous DIEP flaps. Other flap types containing different tissues, such as muscle, have different metabolic rates and may respond differently to both the ischemic insult and the protective effects of RIPC. Additionally, we observed some baseline differences in molecular markers, notably lower caspase 3 and higher p53 levels in the Early RIPC group. While randomization minimises bias, biological variability is inherent in clinical research. Rather than viewing these differences as noise, we interpret them as possible early effects of preconditioning, consistent with molecular priming. Although they complicate interpretation of absolute post‐reperfusion levels, the consistent modulation of markers without baseline differences such as caspase 9 and MLKL supports a genuine protective effect of RIPC.

Our findings provide a mechanistic framework for optimising RIPC in free flap surgery and challenge existing paradigms regarding its mode of action. Early RIPC appears to be more effective than Late RIPC, suggesting that prolonged preconditioning intervals may be required to maximise protective effects. The identification of MLKL as a previously unrecognised target of RIPC warrants further exploration as a marker for flap viability and as a potential pharmacologic target. Furthermore, the limited modulation of oxidative stress in human tissue suggests that additional strategies, such as pharmacologic antioxidants or metabolic interventions, may be necessary to enhance overall cytoprotection in reconstructive surgery. Given the variability in ischemic tolerance across patients, future studies should explore whether patient‐specific factors, including ischemia duration and underlying metabolic conditions, influence the efficacy of RIPC protocols.

## 5 Conclusion

This study provides a mechanistic analysis of RIPC in human free flaps, revealing selective apoptosis and necroptosis modulation while challenging conventional assumptions regarding oxidative stress and survival signalling. The preferential suppression of mitochondrial apoptosis and necroptosis by Early RIPC introduces new directions for translational research and highlights the need for refined conditioning protocols in clinical practice. The unexpected increase in protein nitration suggests a more complex redox adaptation than previously recognised, warranting further investigation into nitric oxide‐related signalling. These insights pave the way for a more targeted and mechanistically informed application of ischemic conditioning in human free flap surgery, with potential implications for refining perioperative management strategies and improving surgical outcomes.

## Author Contributions

**Marius Drysch:** conceptualization (lead), data curation (lead), formal analysis (lead), investigation (equal), methodology (equal), validation (equal), visualization (lead), writing – original draft (lead), writing – review and editing (equal). **Alexander Fiedler:** data curation (equal), formal analysis (equal), methodology (equal), writing – original draft (supporting), writing – review and editing (supporting). **Tabea Kurbacher:** investigation (equal), methodology (equal), visualization (equal), writing – review and editing (equal). **Sonja Verena Schmidt:** funding acquisition (equal), investigation (equal), supervision (equal), validation (equal). **Felix Reinkemeier:** formal analysis (equal), investigation (equal), validation (equal), writing – review and editing (equal). **Flemming Puscz:** formal analysis (equal), funding acquisition (equal), methodology (equal), writing – review and editing (equal). **Mustafa Becerikli:** data curation (equal), resources (equal), supervision (equal), validation (equal). **Maria Fueth:** formal analysis (equal), visualization (equal), writing – review and editing (equal). **Pia Weskamp:** resources (equal), validation (equal), writing – review and editing (equal). **Marcus Lehnhardt:** project administration (equal), resources (equal), supervision (equal), writing – review and editing (equal). **Christoph Wallner:** conceptualization (equal), data curation (equal), supervision (equal), visualization (equal), writing – review and editing (equal). **Alexander Sogorski:** data curation (equal), project administration (equal), resources (equal), supervision (equal), visualization (equal), writing – review and editing (equal).

## Conflicts of Interest

The authors declare no conflicts of interest.

## Data Availability

The data supporting the findings of this study are available from the corresponding author upon reasonable request.
